# Draft genomes of two multidrug-resistant *Enterobacter bugandensis* sp. from African grey parrots in Bangladesh

**DOI:** 10.1128/mra.00414-26

**Published:** 2026-06-22

**Authors:** Mukta Das Gupta, Mishuk Shaha, Mohimanul Islam, Snigdha Das, Plabon Ketan Barua, Uaye Mya, Partha Paul, Ashutosh Das

**Affiliations:** 1 Department of Microbiology and Veterinary Public Health, Faculty of Veterinary Medicine, Chattogram Veterinary and Animal Sciences University 130057, Khulshi, Chattogram, Bangladesh; 2 Genetics, Genomics and Health Research Group, Chattogram Veterinary and Animal Sciences University 130057, Khulshi, Chattogram, Bangladesh; 3 Department of Genetics and Animal Breeding, Faculty of Veterinary Medicine, Chattogram Veterinary and Animal Sciences University 130057, Khulshi, Chattogram, Bangladesh; Loyola University Chicago, Chicago, Illinois, USA

**Keywords:** *Enterobacter bugandensis*, antibiotic-resistant, zoonotic risk, African grey parrots

## Abstract

*Enterobacter bugandensis* (*E. bugandensis*) is a virulent organism, commonly associated with severe, life-threatening infections in neonates. Here, we report the draft genome sequences of multidrug-resistant *E. bugandensis* strains from African grey parrots in Bangladesh. These provide insights into antibiotic-resistant avian-associated isolates and highlight potential zoonotic risks from companion birds in this region.

## ANNOUNCEMENT

Antimicrobial resistance represents a critical challenge to global health. Multidrug-resistant (MDR) *Enterobacter bugandensis* harbors highly transferable antimicrobial resistance genes (1, 2) that can be transferred to other members of the gut microbiota. MDR *E. bugandensis* in the gut of animals and birds may pose a serious threat, as it can spread to humans via food and contaminated water (3); therefore, its presence in captive pet birds, such as African grey parrots, poses a significant public health risk (3, 4).

Between June 2023 and November 2023, we collected cloacal swab samples from captive African grey parrots in Chattogram district, Bangladesh. All protocols applied to animals in this study were approved by the Chattogram Veterinary and Animal Sciences University Ethics Committee (Memo No. CVASU/Dir(R&E) EC/2025/880/6). The samples were then transported and investigated in the Microbiology lab (23.3633°N 91.8039°E) using a standard protocol (5). Briefly, swab samples in buffer peptone water were incubated at 37°C for 24 h, and then cultured overnight on MacConkey agar supplemented with cefotaxime (2 μg/mL) at 37°C. Lactose-fermenting colonies (pink-colored colonies) on MacConkey agar were selected for subsequent characterization. The Kirby-Bauer disk diffusion method (6), following the CLSI M100 breakpoints for Enterobacterales, was used to test the antimicrobial susceptibility of the isolates. For DNA isolation, *E. bugandensis* isolates were cultured on blood agar for 18 h at 37°C. Subsequently, DNA was extracted using a FavorPrep Tissue Genomic DNA Extraction kit (Favorgen Biotech Corporation, Taiwan). DNA libraries were prepared using the Nextera XT DNA Library Preparation Kit (Illumina, San Diego, CA, USA). Genome sequencing was performed on an Illumina NextSeq 2000 platform, generating paired-end reads of 2 × 150 bp. Reads were quality-controlled using Trimmomatic v. 0.39 (7) (sliding window: 4, average quality: 20). The genome of strain 220_1 was assembled with Unicycler 0.5.1 (8) and strain 220_2 with Megahit 1.2.9 (9). The quality of genome assemblies was assessed using Quast v.5.2.0 (10). We used NCBI PGAP v.6.8 (11) for genome annotation. Both isolates exhibited an average nucleotide identity of 98.6% to *E. bugandensis* EB-247 (GenBank accession number NZ_LT992502), as determined by JSpeciesWS using the “blastall” option based on average nucleotide identity (12). Using the GGDC formula 1 (*d_0_*) in the TYGS web-based tool (13), the *in silico* DNA-DNA hybridization score (dDDH) was 94.6% and 94.7%, indicating that the isolates belong to *E. bugandensis*. Both isolates were evaluated for pathogenicity index by PathogenFinder v.1.1 (14), the presence of plasmid by plasmidFinder v.2.0.1 (15), prophages by Phaster web portal (16), antibiotic resistance genes (ARGs) by CARD v.3.2.9 (17) and ResFinder v.4.7.2 (18); virulence genes by VFanalyzer, VFDB 2022 release (19), and siderophores by antiSMASH v.7 (20); bacteriocins by BAGEL4 suite (21); and metabolic functional features by RAST v.2.0 (22). All software used default parameters unless otherwise stated. Genomic characteristics of both *E. bugandensis* isolates (220_1 and 220_2) are presented in Table 1. Both isolates contained 87 predicted virulent genes. They contained 31 predicted ARGs. The phenotypic resistance prediction indicated that both *E. bugandensis* isolates were multidrug-resistant. These findings highlight the complex resistome of avian-associated pathogens. Further in-depth genome mining is warranted to elucidate the molecular mechanisms underlying antibiotic resistance in these avian-associated *E. bugandensis* isolates.

**TABLE 1:** Genome properties of two multidrug-resistant *Enterobacter bugandensis* spp. from African gray parrots in Bangladesh

Strain	220_1	220_2
Biosample accession no.	SAMN55414791	SAMN55414792
GenBank accession no.	JBVDMT000000000	JBVDMS000000000
Total number of reads	11,595,696	11,831,654
Coverage (×)	256×	254×
Genome size (bp)	4,706,363	4,717,967
Completeness of genome assembly	99.88%	99.88%
Sequence types	386	386
No of contigs	36	56
N50 (bp)	370,751	232,950
GC (%)	55.9	55.9
Genes (total), PGAP	4,516	4,542
Pseudo genes (total)	55	57
CDSs (total)	4,429	4,435
CDS with protein	4,374	4,378
CDSs (without protein)	55	57
rRNA:	1, 3, 3 (5S, 16S, 23S)	10, 8, 4 (5S, 16S, 23S)
tRNA:	73	78
Number of RNAs	79	96
Number of subsystems	374	374
Predicted virulence genes (VFGs)	87	87
No. of prophages	6	3
Bacteriocins	Colicin, bottromycin	Colicin, bottromycin
Mobile genetic elements (MGEs)	306	308
Plasmids	No plasmid	No plasmid
Predicted antibiotic genes (ARGs) in CARD	31	31
Predicted resistance against antibiotic families	Amoxicillin, amoxicillin + clavulanic acid, ampicillin, ampicillin + clavulanic acid, cefotaxime, cefoxitin, ceftazidime, piperacillin, piperacillin + tazobactam, ticarcillin, ticarcillin + clavulanic acid, fosfomycin, ciprofloxacin, nalidixic acid, trimethoprim, chloramphenicol.	Amoxicillin, amoxicillin + clavulanic acid, ampicillin, ampicillin + clavulanic acid, cefotaxime, cefoxitin, ceftazidime, piperacillin, piperacillin + tazobactam, ticarcillin, ticarcillin + clavulanic acid, fosfomycin, ciprofloxacin, nalidixic acid, trimethoprim, chloramphenicol.
Predicted siderophores	NRP-metallophore, NRPS, NRPS-independent, IucA/IucC-like siderophores, (Thio)azol(in)e-containing peptides, linear and macrocyclic butyrolactone, terpene-precursor, and aryl polyene.	NRP-metallophore, NRPS, NRPS-independent, IucA/IucC-like siderophores (Thio)azol(in)e-containing peptides, linear and macrocyclic butyrolactone, terpene-precursor, and aryl polyene

## Data Availability

The genome sequences are available under BioProject accession number PRJNA1425463, with individual accession numbers as provided in Table 1.
